# EMC1 Is Required for the Sarcoplasmic Reticulum and Mitochondrial Functions in the *Drosophila* Muscle

**DOI:** 10.3390/biom14101258

**Published:** 2024-10-05

**Authors:** Carlos Antonio Couto-Lima, Maiaro Cabral Rosa Machado, Lucas Anhezini, Marcos Túlio Oliveira, Roberto Augusto da Silva Molina, Rodrigo Ribeiro da Silva, Gabriel Sarti Lopes, Vitor Trinca, David Fernando Colón, Pablo M. Peixoto, Nadia Monesi, Luciane Carla Alberici, Ricardo Guelerman P. Ramos, Enilza Maria Espreafico

**Affiliations:** 1 Department of Cell and Molecular Biology, Faculty of Medicine of Ribeirão Preto, University of São Paulo (FMRP-USP), Ribeirão Preto 14049-900, SP, Brazil; 2 Department of Biotechnology, College of Agricultural and Veterinary Sciences, Sao Paulo State University, Jaboticabal 14884-900, SP, Brazil; 3 Cellular and Molecular Biology Program, Faculty of Medicine of Ribeirão Preto, University of São Paulo (FMRP-USP), Ribeirão Preto 14049-900, SP, Brazil; 4 Institute of Biological Sciences and Health, Federal University of Alagoas, Maceió 57072-900, AL, Brazil; 5 Department of Pharmacology, Ribeirão Preto Medical School, University of São Paulo, Ribeirão Preto 14049-900, SP, Brazil; 6 Baruch College and Graduate Center, The City University of New York, New York, NY 10010, USA; 7 Department of Clinical Analyses, Toxicology and Food Science, School of Pharmaceutical Sciences of Ribeirão Preto, University of São Paulo, Ribeirão Preto 14040-903, SP, Brazil; 8 Department of Biomolecular Sciences, School of Pharmaceutical Sciences of Ribeirão Preto, University of São Paulo, Ribeirão Preto 14049-900, SP, Brazil

**Keywords:** endoplasmic reticulum membrane protein complex, mitochondria, musculature

## Abstract

EMC1 is part of the endoplasmic reticulum (ER) membrane protein complex, whose functions include the insertion of transmembrane proteins into the ER membrane, ER–mitochondria contact, and lipid exchange. Here, we show that the *Drosophila melanogaster EMC1* gene is expressed in the somatic musculature and the protein localizes to the sarcoplasmic reticulum (SR) network. Muscle-specific *EMC1* RNAi led to severe motility defects and partial late pupae/early adulthood lethality, phenotypes that are rescued by co-expression with an *EMC1* transgene. Motility impairment in EMC1-depleted flies was associated with aberrations in muscle morphology in embryos, larvae, and adults, including tortuous and misaligned fibers with reduced size and weakness. They were also associated with an altered SR network, cytosolic calcium overload, and mitochondrial dysfunction and dysmorphology that impaired membrane potential and oxidative phosphorylation capacity. Genes coding for ER stress sensors, mitochondrial biogenesis/dynamics, and other EMC components showed altered expression and were mostly rescued by the *EMC1* transgene expression. In conclusion, EMC1 is required for the SR network’s mitochondrial integrity and influences underlying programs involved in the regulation of muscle mass and shape. We believe our data can contribute to the biology of human diseases caused by *EMC1* mutations.

## 1. Introduction

*EMC1* mutations have been associated with severe neurological, muscular, and skeletal abnormalities in children [1,2,3]. The *EMC1* gene is evolutionarily conserved and ubiquitously expressed in all eukaryotes. Its orthologue, *YCL045C*, was first characterized in *Saccharomyces cerevisiae*, in which it was proposed to encode a component of the endoplasmic reticulum (ER) membrane protein complex (EMC), required for efficient folding of transmembrane proteins in the ER [4]. Subsequently, mammalian EMC was shown to encompass 10 subunits (EMC1–10) and to interact with the ER-associated degradation (ERAD) proteins UBAC2 and Derlin-2, suggesting a close link between EMC and ERAD [5]. Using *in vitro* assays, proteomics, cultured mammalian cells, as well as *Drosophila melanogaster* photoreceptors and eye imaginal discs as models, several groups [5,6,7,8,9,10,11] showed that EMC is essential for the biogenesis of many multi-pass transmembrane and tail-anchored proteins. Consistent with this idea, the biosynthesis of the *Caenorhabditis elegans* acetylcholine receptors is also dependent on EMC function [12]. High-throughput protein–protein interaction studies in several models provided evidence for the interaction of EMC1 and other EMC components with mitochondrial and cytoskeleton proteins, transcription factors, and components of the cellular secretory pathway [13], in agreement with the idea that EMC is involved in vesicle trafficking [14]. In summary, EMC may play a crucial role in membrane protein biogenesis by integrating various essential functions. These include stabilizing transmembrane proteins, recruiting folding factors, and shielding folding intermediates from recognition and degradation by the ER quality control machinery [15].

Although the pathogenesis of EMC1 mutations has been associated with glial dysfunction [2], the clinical phenotypes are relatively broad and the roles of EMC in other organs cannot be discarded. Here, we describe phenotypical and functional defects caused by EMC1 depletion in the somatic musculature of *D. melanogaster*. Proper muscle contraction and relaxation cycles require a dense network of sarcoplasmic reticulum (SR) and mitochondria in close proximity to the myofibrils, as well as an extensive contact surface between these organelles. This is important for the regulation of mitochondrial ATP production via Ca^2+^ from the SR [16]. The contact surface is also a major site of lipid biosynthesis and lipid exchange between the two organelles, playing a pivotal role in the maintenance of their structure [17]. Remarkably, a study in *S. cerevisiae* aimed at identifying genes required for the exchange of phospholipids between ER and mitochondria found that EMC mutants show a deficient transfer of phosphatidylserine to mitochondria, associated with reduced ER–mitochondrial tethering, leading to the hypothesis that the EMC complex is required for both functions [18,19]. Our results demonstrate that muscle-specific EMC1 knockdown causes defective locomotion, decreased viability, and a shortened life span, which are associated with muscular deformities, a deranged SR network, cytosolic Ca^2+^ overload, and impaired mitochondrial oxidative phosphorylation. Our results suggest that EMC defects may also lead directly to the muscular phenotypes seen in human patients with EMC1 mutations.

## 2. Materials and Methods

### 2.1. Fly Strains and Genetics 

The following fly lines were used: *UAS-EMC1-RNAi*/*TM3* (VDRC #V8477); *UAS-EMC1-RNAi* and *Mef2-GAL4* (BDSC #34581 and #27390, respectively); *UAS-mitoGFP*/*CyO*; *MKRS*/*TM6B* (kindly provided by Dr. Cheng-Yu Lee, Life Sciences Institute, University of Michigan); and *w^1118^*. The flies were cultured at 25 °C with a 12:12 h light/dark cycle and maintained on a standard diet (0.5% agar, 1.5% yeast, 2.5% sucrose, 8.5% corn extract, 5% dextrose, 0.5% Nipagin, and 0.5% acid mix [8.2% propionic acid and 45.8% phosphoric acid]).

### 2.2. Generation of UAS-EMC1 Transgenic Fly Lines 

The *Drosophila EMC1* cDNA (3286 bp)—spanning the entire coding region (2748 pb) plus 117 bp of the 5′UTR and 127 pb of the 3′UTR (LD19064, ID BT031325.1), cloned in the pBlueScript plasmid—was obtained from the Berkeley *Drosophila* Genome Project “http://flybase.org/reports/FBcl0153403.html (accessed on 13 April 2022)”. The cDNA was subcloned into the EcoRI site of the pUAST vector [20] and sequenced for confirmation. Midiprep IlustraTM Plasmid prep Midflow Kit (GE Healthcare Life Sciences, Marlborough, MA, USA) was performed to obtain quantities of DNA suitable for microinjection. P-element transformation was carried out according to standard procedures [21]. The *UAS-EMC1* construct (final concentration 0.5 μg/μL) was injected into *D. melanogaster y^−^w^−^* embryos, together with the phsπ helper plasmid (final concentration 0.1 μg/μL) [22]. The injection was performed using a Leica inverted microscope and an Eppendorf FemtoJet microinjector. Injected embryos were kept in a moist chamber at 18 °C for 2 days, when then the larvae were transferred to fly food vials and cultured at 25 °C (~20–30 per vial). G0 flies were mated individually with the parental strain and the resulting independent F1 transgenic progeny were used to establish homozygous and/or balanced lines. Five independent UAS-EMC1 lines were created and named according to genetic tracking, 18R, 18.4, and 22.2, which were inserted on chromosome 2, and the lines 27R and 27.1, which were inserted on chromosomes 2 and 3.

### 2.3. Longevity, Locomotor, and Feeding Assays 

For longevity, 50 adult flies of each genotype, separated into 5 groups, were maintained in standard diet vials. The flies were transferred to new vials every 2 days and the number of dead flies was then recorded. The data are presented as Kaplan–Meier survival distributions.

Adult fly locomotion was quantitated by climbing assays. Using a countercurrent apparatus [23], three hundred males per age group (0–6 h, 1, 3, and 4 day-old) and per genotype were placed into the first chamber, taped to the bottom, then given 20 s to climb a distance of 10 cm. Flies that successfully climbed 10 cm or beyond in 20 s were shifted to a new chamber, and both sets of flies were given another opportunity to climb the 10 cm distance. This procedure was repeated a total of five times. To quantify the performance, flies received a score as follows: the percentage of the cohort in successive tubes 6 to 1 was multiplied, respectively, by 1, 0.8, 0.6, 0.4, 0.2, and 0. Scores were totaled for each round and the mean and standard error were calculated based on a total of twelve rounds. Statistical analysis used one-way ANOVA/GraphPad Prism Version 8.3.0 for Mac (GraphPad Software, La Jolla, CA, USA).

Larval locomotion was quantitated by crawling assays. A single larva was selected, washed briefly in distilled water, transferred to the center of a fresh bacterial agar plate, and allowed to recover for about 20 s. After this time, the larval path on the agar plate was monitored during 120 s. Petri dishes were set on top of square millimeter graph paper. Plates were photographed, paths were measured, and the data were processed using ImageJ software (version 1.53) [24].

For feeding assays, adult flies of the appropriate genotypes were kept in empty culture vials for 2 h and then placed in vials containing standard diet with the addition of 2.5% (w/v) Brilliant Blue (Sigma-Aldrich, St. Louis, MI, USA). The animals were maintained on this diet for 4 h. The flies were visualized under a stereomicroscope. The intestines were dissected in 1X PBS and immediately photographed.

### 2.4. Nucleic Acid Extraction and Quantitative Real-Time PCR

For DNA extraction, the tissues were homogenized in 300 µL of ice-cold lysis buffer (75 mM NaCl, 25 mM EDTA, 25 mM HEPES, pH 7.5), followed by the addition of 30 µL of 10% SDS and 2.5 μL of 20 mg/mL proteinase K, then incubated for 30 min at 37 °C. Three hundred microliters of phenol–chloroform–isoamyl alcohol (25:14:1) were then added and mixed by inversion, and the tube was incubated on ice for 2 min, followed by centrifugation at 2000× *g* for 10 min at 4 °C. The aqueous phase was transferred to a new tube, and the procedure was repeated once. Nucleic acids were precipitated by adding 1 mL of ice-cold 100% ethanol and incubating overnight at −20 °C. The samples were then centrifuged at 16,000× *g* for 20 min at 4 °C and washed with ice-cold 70% ethanol. The pellets were dried and resuspended in 20 μL of TE buffer (10 mM Tris/HCl, 0.1 mM EDTA, pH 7.5), and the final DNA concentration was estimated by absorbance at 260 nm.

Total RNA was extracted by tissue homogenization in the presence of 300 µL RNAzol^®^ RT (Molecular Research Center), followed by the addition of 120 µL DEPC water, vortexing for 15 s, then incubation at room temperature for 15 min. The samples were centrifuged for 15 min at 12,000× *g*, and the supernatant transferred to a new tube. Three hundred microliters of ice-cold isopropanol was added, and the samples were centrifuged as previously. The RNA pellet was washed twice with 500 µL ice-cold 75% ethanol, centrifuged at 8000× *g* for 3 min, resuspended in 30 µL RNase-free water, and stored at −80 °C. The iScript™ cDNA Synthesis Kit (Bio-Rad Laboratories, Hercules, CA, USA) and 1 µg of total RNA were used for cDNA synthesis, according to the manufacturer’s specifications.

Quantitative real-time PCR experiments were performed on the StepOne Plus equipment (PE Applied Biosystems, Foster City, CA, USA) using the GoTaq^®^ qPCR Master Mix (Promega, Madison, WI, USA), according to the manufacturer’s recommendation. The primers used for the RT-qPCR analysis are described in Table 1. Five thoraces were utilized for each biological replicate, and biological triplicates were conducted for each of the RT-qPCR experiments.

### 2.5. Preparation of Anti-EMC1 Antibody and Immunoblot Analyses

We amplified a 1370 bp fragment corresponding to the C-terminal region of EMC1. Forward and reverse primers containing cleavage sites for the restriction enzymes EcoRV and HindIII were used (F-GATATCGTAAAACCAGCATCTTGTGGAGG and R-AAGCTTCTATTTCCACGCCTGCTTG). The amplified fragment was subcloned into the TOPO^®^ vector and then cloned into the pQE81^®^ expression vector. BL-21 strain *E. coli* bacteria, containing the pQE81 vector with the insert, were cultured in liquid LB medium until reaching an OD600 of 0.6. Expression of the recombinant protein was induced by adding 100 mM IPTG and maintained for 3 h at 37 °C. After induction, the bacteria were centrifuged, resuspended, and homogenized in phosphate buffer with the addition of Triton X-100 and protease inhibitors. The homogenate was sonicated, and after further centrifugation, the supernatant was applied to a nickel column (His TRAP FF 1 ml column—GE) using FPLC (Äkta purifier, Uppsala, Sweden). The resin was equilibrated, washed, and the protein was eluted using an imidazole gradient, with the eluate collected in 1 mL fractions. Subsequently, 200 µg of recombinant protein was emulsified in Freund’s adjuvant (1:1) and injected subcutaneously into rabbits. Boosts were administered with 100 µg of antigen emulsified in adjuvant, followed by two additional boosts given at intervals of 4 to 6 weeks. Serum samples were collected 10 to 14 days after the last inoculation. Blood was allowed to clot for 30 to 60 min at 37 °C, after which the clot was removed and the serum was centrifuged at 10,000× *g* for 10 min at 4 °C. Sodium azide (final concentration of 0.2 mM) was added to the supernatant, and aliquots were stored at −20 °C for later purification of antibodies using nitrocellulose strips.

The tissues were homogenized in lysis buffer (0.1% Triton-X100, 10 mM EDTA, 1 mM DTT, 100 mM KCl, 20 mM HEPES (pH 7.5), 5% glycerol, 1 μg/mL chymostatin, 1 μg/mL leupeptin, 1 μg/mL pepstatin A, and 1 mM PMSF). The lysates were subsequently clarified by centrifugation and the supernatant recovered and stored for further analyses. The proteins were separated on 12% SDS-PAGE gels and transferred to nitrocellulose membranes (Millipore, St. Louis, MO, USA), which were then blocked in 5% powder milk in 1X PBS and incubated with the primary anti-EMC1 antibody (1:1000) before incubation with the HRP-conjugated anti-rabbit secondary antibody (1:1000). Antibody complexes were visualized by enhanced chemiluminescence (ECL). The results are an average of at least three biological replicates per genotype for all reported data.

### 2.6. Immunostaining of Embryo, Larvae, and Adult Musculature

Embryos were staged, fixed, and labeled with Rhodamine phalloidin (1:1000, #R415 ThermoFisher, Waltham, MA, USA) as described by [31]. The muscle wall of wandering third-instar larvae or adult hemi-thoraces were dissected in 1X PBS, permeabilized, fixed with 4% paraformaldehyde, and stained as previously described [32,33]. Incubation with the primary antibody was performed overnight and with the secondary antibody for 4 h. For EMC1 staining, we used the affinity-purified rabbit anti-EMC1 polyclonal antibody (15 to 20 μg/mL) described above. For Calreticulin staining, chicken IgY anti-Calreticulin (1:300 #Abcam/ab2908) was used. Image acquisition was performed using either Leica SP5 or Zeiss LSM780-AxioObserver confocal microscopes. The anti-EMC1 antibody was detected with the secondary anti-rabbit IgG-Alexa Fluor-594. The anti-Calreticulin was detected with anti-chicken IgY-Alexa Fluor-633. Myofibrils were revealed using Rhodamine phalloidin (1:1000, #R415 ThermoFisher). Measurements were obtained from 36 external transverse muscles and 12 dorsal external oblique muscles. The number of nuclei in muscles 6 and 7 was calculated as mean ± S.D. for *n* = 35 muscles of each genotype.

### 2.7. Calcium Mobilization Assay and Measurements of Mitochondrial Membrane Potential and Respiration

One hundred thoraces of 2-day-old flies were dissected in DMEM medium, without phenol. For each group, a total of a hundred thoraces were dissected and distributed into 5 wells (20 thoraces of each group/well) of a 96-well Black plate (Corning^®^ 96 Well Flat Clear Bottom Black), followed by incubation with Fluo-4AM (4 mM) or TMRE [34] in Hanks solution containing 1.3 mM CaCl_2_, for 50 min, at 37 °C and 5% CO_2_. Thoraces were then washed 5 times for 10 min each wash. Recording of Fluo-4 fluorescence or TMRE was carried out on a FlexStation 3 Benchtop Multi-Mode Microplate Reader, using the equipment’s automatic settings. 

Mitochondrial respiration assays were performed at 25 °C using the high-resolution Oxygraph-2k (Oroboros Instruments, Innsbruck, Austria), equipped with the DatLab software package for data acquisition and analysis. Six adult thoraces were dissected and incubated in 2.1 mL MiR05 respiration buffer (20 mM HEPES, 10 mM KH_2_PO_4_, 110 mM sucrose, 20 mM taurine, 60 mM K-lactobionate, 0.5 mM EGTA, 3 mM MgCl_2_, 1 g/L fatty acid-free BSA, pH 7.1) containing 2 mM of each respiratory substrate (1.5 mM pyruvate, 0.28 mM malate, 1.3 mM glutamate), and the chamber was closed to obtain the Leak (n) respiratory state. Then, it was added sequentially to 240 μM ADP, 0.5 μg/mL oligomycin, and 0.625 μg/mL antimycin A to obtain, respectively, the OXPHOS, Leak (Omy) and non-mitochondrial respiration rates.

### 2.8. Electron Microscopy Analyses

Thoraces of newly eclosed and 2-day-old animals were dissected and fixed in 3% glutaraldehyde in 0.1 M cacodylate buffer (pH 7.2–7.4) at 4 °C for 6 h. Then, 1% osmium tetroxide was added as a post-fixative (4 °C, 1.5 h). The samples were then immersed in serial dilutions of ethanol (50, 70, 90, 95, and four times 100%, each for 15 min) and acetone (for 30 min) for dehydration. The fixed thoraces were embedded in epoxy resin (Epoxy Embedding Medium Kit, Sigma, MI, USA) and sliced in semi-thin and ultra-thin sections using an ultramicrotome. The ultra-thin sections were examined using a Jeol 100-CXII electron microscope (Jeol Ltd., Tokyo, Japan). The images were recorded using an ORCA-HR digital camera (Hamamatsu Photonics, Shizuoka, Japan).

## 3. Results

### 3.1. EMC1 Knockdown in the Drosophila Musculature Triggers Muscle Deformation and Severe Motility Defects

We first cloned, expressed in bacteria, and affinity-purified the C-terminal half (aa 460-915) of the *Drosophila* EMC1 protein fused with a 6xHis-tag (Supplementary Figure S1A). We generated a polyclonal antibody against the fusion protein, which was able to detect a polypeptide of ~100 kDa, consistent with the predicted molecular weight of the fly EMC1 protein (Supplementary Figure S1B). We have also observed by quantitative PCR that *EMC1* transcript levels throughout development (Supplementary Figure S1C) are in agreement with the expression profiles determined by high-throughput RNAseq data [35].

**Figure 1:**
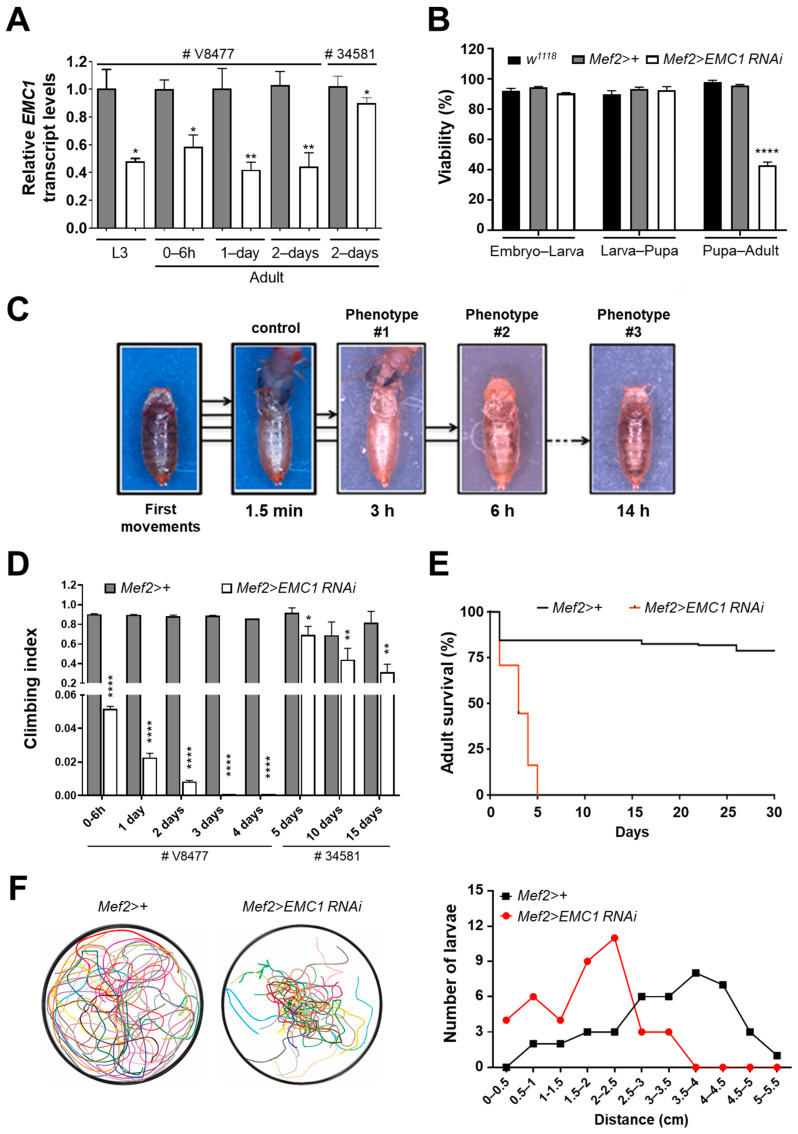
RNAi-mediated silencing of *EMC1* in the *Drosophila* muscle leads to pupal lethality and severe motor disabilities in adults and larvae. (**A**) *EMC1* transcript levels were calculated as described in the Section 2 for individuals of the indicated genotype, using either the VDRC RNAi line V8477 or the BDSC RNAi line 34581. Transcripts were normalized by the levels of *EMC1* in the *Mef2>+* control at the indicated developmental time. (**B**) Viability was calculated as the percentage of individuals of the indicated genotype that reached the next developmental stage. (**C**) The adult eclosion process is represented from frames of movies recorded for *Mef2>+* control and *Mef2>EMC1 RNAi* V8477 individuals with various degrees of phenotype severity (#1, #2, and #3). The initial time represents the first adult movements still inside the puparium, until the animals leave the pupal case (WT and phenotype #1) or notably fail to do so (#2 and 3). See details in Videos S1A–C. (**D**) Locomotion of adult individuals of the indicated genotypes and ages was evaluated as the average (±S.D.) climbing abilities. *, **, and **** indicate, respectively, *p* < 0.05, *p* < 0.01, and *p* < 0.0001, according to Student’s *t*-tests applied between *Mef2>+* controls and *Mef2>EMC1 RNAi* animals. Adult life span (**E**) and larval locomotion (**F**) analyses were performed as described in the Section 2 using *Mef2>+* controls and *Mef2>EMC1 RNAi* V8477 individuals. Individual larval crawling paths are represented in different colors in (**F**) (left panel).

To test the physiological roles of muscular EMC, we crossed two *UAS-EMC1-RNAi* lines with the muscle-specific *Mef2-Gal4* driver line. The efficiency of *EMC1* knockdown was ~50% for the VDRC line V8477 and only ~15% for the BDSC line 34581 (Figure 1A). Nevertheless, visible locomotor phenotypes were observed for both lines. Most *EMC1*-silenced animals of the *Mef2>EMC1-RNAi* V8477 genotype underwent normal development and complete metamorphosis, reaching the late pupal stage, but ~60% of the adults failed to eclose (Figure 1B,C, Video S1). In the *Mef2>+* control flies, eclosion was a rapid process that concluded in about 1.5 min (Figure 1C, Video S1A). In the *Mef2>EMC1-RNAi* V8477 flies, of the animals that did eclose, one-third died halfway during exit of the pupal case (Figure 1C, Video S1C), and for the other two-thirds, the eclosion process took much longer than in controls (Figure 1C, Video S1B). The peristaltic abdominal and leg movements of the partially emerged animals were uncoordinated, slow, irregular, and incapable of exerting effective force against either the pupal case wall or the outside plate surface (Video S1C). The adults that succeeded exiting the puparium exhibited slow and wobbly locomotion, failed to fly and to climb (Figure 1D, Video S2), and had a very short lifespan (Figure 1E). Moreover, we frequently observed that eclosed adults were unable to fully ingest food and had several wing-related problems (Supplementary Figure S1D–F); however, these phenotypes were not investigated any further. Motility defects were also present during the larval stage of the *Mef2>EMC1-RNAi* V8477 animals (Figure 1F), suggesting an intrinsic failure of the somatic muscle function in *EMC1*-silenced flies. Although *EMC1* knockdown in animals of the *Mef2>EMC1-RNAi* 34581 genotype was mild, the motility of adult flies was also impacted in an age-dependent manner (Figure 1D). Because *EMC1* knockdown in these flies was much less efficient, from this point on in the paper, we solely used *Mef2>EMC1-RNAi* V8477 animals and are herein referring to them only as *Mef2>EMC1-RNAi*. Our results show that muscular EMC1 is important for larval and adult locomotion.

**Figure 2:**
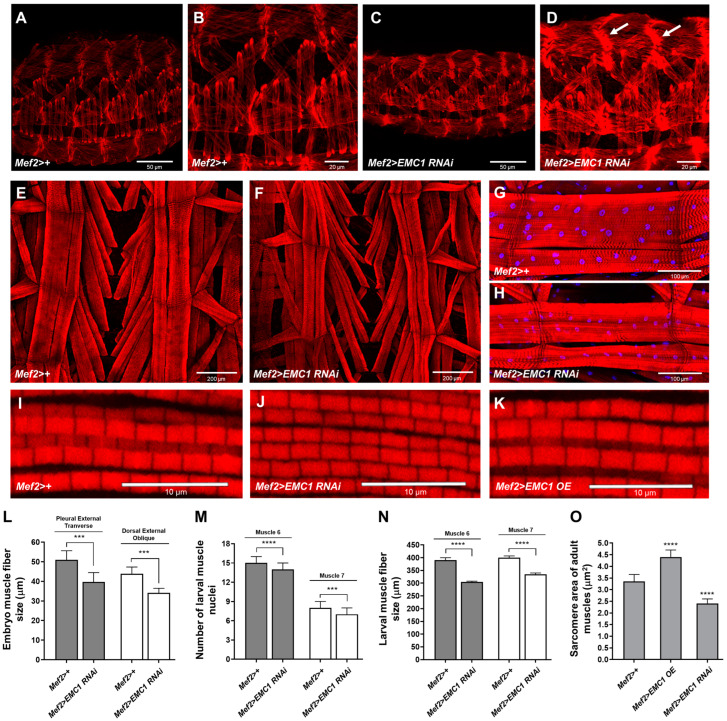
*EMC1* knockdown causes muscle deformations and reduced size. Immunofluorescent confocal microscopy images of representative musculatures of stage 16 embryos (**A**–**D**), third-instar larvae (**E**–**H**), and 2-day-old adults (**I**–**K**) of the indicated genotypes. Samples were treated with rhodamine-phalloidin and DAPI to stain F-actin fibers and nuclei, as described in the Section 2. Images in (**B**,**D**) are enlargements of (**A**,**C**), respectively. Arrows indicate the zones of fiber insertion with a strong F-actin staining in *Mef2>EMC1 RNAi* animals. (**L**–**O**) Quantification of data in (**A**–**K**) as averages ± S.D. *** and **** represent, respectively, *p* < 0.001 and *p* < 0.0001, according to Student´s *t*-tests.

We next evaluated if *EMC1* silencing caused alterations in muscle size and organization. Immunofluorescent confocal imaging shows that depletion of EMC1 in late-stage embryos causes distortions in muscle fiber organization, with myofilaments exhibiting a reduced size and a general loss of tonus (Figure 2A–D,L). Also, the zones of fiber insertion appear dislodged and with a strong F-actin staining (Figure 2D, white arrows). In the larva, the musculature of segments A3–6 had shorter, thinner, and distorted muscle fibers as well as reduced nuclei size and number (Figure 2E–H,M,N). In the indirect flight muscles of eclosed adults, we observed a decrease in the thickness of the myofibrils and in the sarcomere area (Figure 2I,J,O). These observations could explain the severe locomotor phenotype of the *Mef2>EMC1-RNAi* animals described above.

We next generated transformant fly lines containing the full-length *EMC1* coding sequence downstream of the Gal4-responsive *UAS* element. We obtained five independent transformants, four of which showed high levels of *EMC1* transcripts when crossed with *Mef2-Gal4* (Supplementary Figure S2A). We then performed a sequence of crosses using transformant line #18R to restore the levels of EMC1 in *EMC1*-silenced animals. The *Mef2>EMC1-OE;EMC1-RNAi* animals had more than a 100-fold increase in transcript levels; protein levels were approximately the same as in the *Mef2>+* controls, although *Mef2>EMC1-OE* showed EMC1 protein levels 6–8-fold higher (Supplementary Figure S2B,C). We observed in *Mef2>EMC1-OE;EMC1-RNAi* animals a partial rescue of adult eclosion, locomotion, and life span (Supplementary Figure S2D,E, Videos S3 and S4). *Mef2>EMC1-OE* animals were not significantly different than controls, except for a larger body size (Supplementary Figure S2F,G) and larger sarcomere area of the adult indirect flight muscles (Figure 2K,O), features that were not further investigated.

### 3.2. EMC Is Required for Maintenance of the Sarcoplasmic Reticulum Network and Cytosolic Calcium Homeostasis

**Figure 3:**
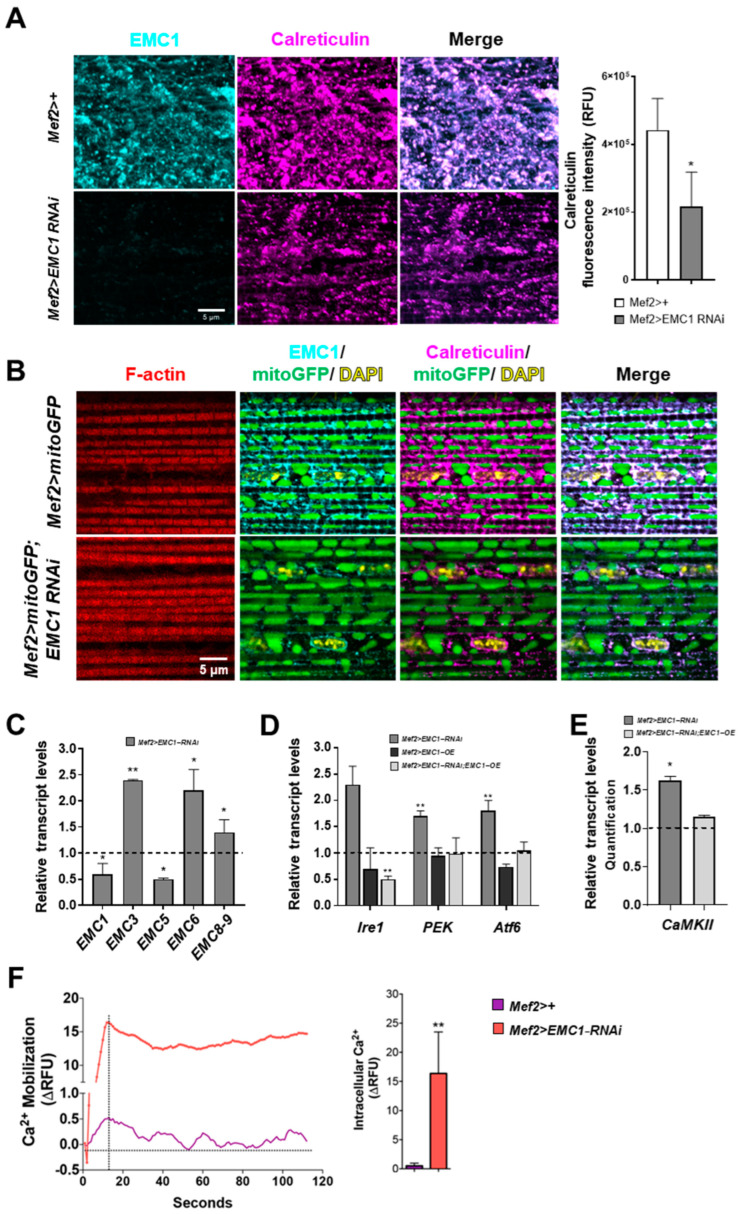
EMC1 is required for maintenance of the sarcoplasmic reticulum network and cytosolic calcium homeostasis. Immunofluorescent confocal microscopy images (Z-stacks) of representative indirect flight muscles of *Mef2>+* and *Mef2>EMC1*−*RNAi* adults stained with anti−EMC1 and anti-calreticulin; right panel, average (±S.D.) level of calreticulin relative fluorescence intensity. (**A**), and of *Mef2>mitoGFP* and *Mef2>mitoGFP;EMC1*−*RNAi* adults stained with anti-EMC1, anti-calreticulin, rhodamine-phalloidin, and DAPI (**B**). (**C**–**E**) Transcript levels of the indicated genes were calculated as described in the Section 2 for 2-day-old adult individuals of the indicated genotypes, normalized by the levels in the *Mef2>+* control. (**F**) Left panel, representative real-time Fluo−4 AM measurements of calcium mobilization in the thoracic muscle of 2-day-old adults of the indicated genotypes; right panel, average (±S.D.) level of intracellular calcium at the timepoint of signal saturation (indicated by a vertical dashed line on the left panel). ΔRFU, change in relative fluorescence units. * and ** represent, respectively, *p* < 0.05 and *p* < 0.01, according to Student´s *t*-tests applied between the *Mef2>+* controls and *Mef2>EMC1*−*RNAi*, *Mef2>EMC1*−*OE* or *Mef2>EMC1*−*OE;EMC1-RNAi* animals. For RT−qPCR experiments, five thoraces were used for each biological replicate, with a total of three replicates utilized per phenotype and one hundred thoraces from 2-day-old flies were divided into five groups for the calcium experiments.

To further investigate the cellular alterations underlying the locomotion defects and muscle morphology alterations in the absence of EMC1, the SR network of the indirect flight muscles of adult thoraces was analyzed using confocal microscopy imaging. We confirm that EMC1 colocalizes with the SR marker Calreticulin along the whole dense SR network of the musculature (Figure 3A,B). A faint staining of EMC1 was still observed in the muscle fibers of *Mef2>EMC1-RNAi* animals, which in turn also presented a remarkable decrease in Calreticulin staining and a sparse SR network (Figure 3A,B). In *EMC1*-silenced individuals trapped in the pupal case 1 day passed eclosion, the images reveal stronger Calreticulin staining, with an uneven distribution of the SR network (Supplementary Figure S3A).

We also checked the transcript levels of other members of the EMC complex in the thoracic muscles of *Mef2>EMC1-RNAi* animals, and observed an increase for *EMC3*, *EMC6*, and *EMC8–9* and decrease for *EMC5* (Figure 3C), indicating a general disarrangement of the complex. We also observed an increase in the transcript levels of major sensors for the ER unfolded protein response, *Ire1*, *PEK*, and *Atf6*, which returned to normal levels in *Mef2>EMC1-OE;EMC1-RNAi* animals (Figure 3D). Because of the known role of the SR in regulating intracellular Ca^2+^ concentration, we used Fluo-4 fluorescence to determine the cytosolic-free Ca^2+^ levels. EMC1-silenced thoraces have cytosolic-free Ca^2+^ >15-fold higher than the control (Figure 3F). This is consistent with the increase in transcript levels of the Ca^2+^-calmodulin-regulated *CaMKII* in *Mef2>EMC1-RNAi* animals, which also return to normal levels in *Mef2>EMC1-OE;EMC1-RNAi* animals (Figure 3E). Altogether, these results suggest that proper EMC function is required for normal SR network organization and function as well as regulation of intracellular Ca^2+^ levels in the *Drosophila* musculature.

### 3.3. Altered Mitochondrial Shape and Respiration in EMC1-Silenced Fly Muscles

**Figure 4:**
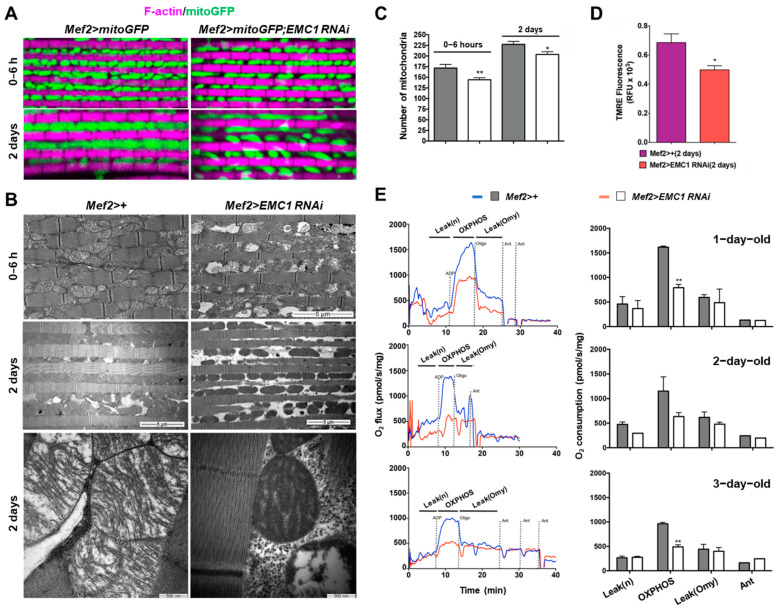
*EMC1* silencing results in severely altered mitochondrial morphology, lower membrane potential, and lower oxidative phosphorylation in adult indirect flight muscles. Immunofluorescent confocal microscopy images (**A**) and transmission electron microscopy of longitudinal sections (**B**) of representative thoracic indirect flight muscles of adult flies of the indicated genotype and age. Samples were stained with rhodamine-phalloidin in (**A**). (**C**) Quantification of intermyofibrillar mitochondria data shown in (**A**,**B**). (**D**) TMRE signal from isolated thoraces of 2-day-old adults was obtained through fluorescence reading using the FlexStation 3 Benchtop Multi-Mode Microplate Reader, indicating the genotype and showing relative mitochondrial membrane potential. RFU, relative fluorescence units. (**E**) Representative traces (left panels) and quantification (right panels, average ±S.D.) of oxygen consumption rates of 1- to 3-day-old adult thoraces. Dissected tissues were incubated in the Mir05 respiration buffer in the presence of pyruvate, malate, and glutamate (Leak (n) respiratory state), followed by addition of ADP (OXPHOS respiratory state), oligomycin (Leak (Omy) respiratory state), and finally antimycin A (Ant, non-mitochondrial respiration), as described in the Section 2. * and ** represent, respectively, *p* < 0.05 and *p* < 0.01, according to Student´s *t*-tests applied between the *Mef2>+* controls and *Mef2>EMC1-RNAi* animals.

Given the importance of the SR–mitochondria relationship and the sensitivity of mitochondria to Ca^2+^ levels, we investigated possible changes in mitochondrial morphology in *Mef2>EMC1*−*RNAi* muscles using a mitochondrially tagged GFP marker (mitoGFP). In the indirect flight muscles of *Mef2>+* adults, as the flies age from 0–6 h to 2 days post-eclosion, mitochondria almost fully occupy the intermyofibrillar spaces (Figure 3B and Figure 4A). Ultrastructural analyses using transmission electron microscopy show large mitochondria with highly developed cristae (Figure 4B). In *Mef2>EMC1*−*RNAi* animals, the intermyofibrillar spaces are not highly occupied by mitochondria, having a significant portion of translucent areas (Figure 4A,B). We observed alterations in the mitochondrial size, structure, abundance, and distribution in these animals, including a combination of atypically large and atypically small organelles as well as the presence of mitochondria with intermyofibrillar projections (Figure 3B and Figure 4A and Supplementary Figure S3A). In addition, they are heterogeneous in size and highly electron-dense in appearance (Figure 4B). *EMC1* silencing caused a slight decrease in the number of identified mitochondria (Figure 4C), which was accompanied by a ~30% decrease in the mitochondrial DNA copy number (Supplementary Figure S3B). Transcript levels of major components of the mitochondrial biogenesis and dynamics processes, such as *spargel*, *Marf*, *Pink1*, and *parkin*, were ~2-fold upregulated and *Drp1* was dramatically downregulated in 0–6-hour-old flies; in 2-day-old flies, all genes were downregulated compared to controls (Supplementary Figure S3C). In *EMC1*-silenced individuals trapped in the pupal case 1 day passed eclosion, the levels of these transcripts were similar to 0–6-hour-old flies that did eclose (Supplementary Figure S3D). Deregulation in the level of these transcripts was not fully restored in *Mef2>EMC1*−*OE;EMC1*−*RNAi* animals (Supplementary Figure S3E), although these flies did not show significant changes in mitochondrial morphology and organization, as judged by confocal microscopy analyses (Supplementary Figure S3F).

We next checked if *EMC1* silencing and the associated changes in mitochondrial morphology would disturb mitochondrial membrane potential and oxygen consumption. We observed a 25% decrease in TMRE fluorescence in 2-day-old *Mef2>EMC1-RNAi* thorax samples (Figure 4D), indicating compromised mitochondrial membrane potential. Using high-resolution respirometry, we monitored mitochondrial oxygen consumption of dissected thorax muscles from 1- to 3-day-old flies and observed that EMC1 depletion causes a ~50% drop in oxidative phosphorylation (OXPHOS) at all ages (Figure 4E). The other respiratory states analyzed, Leak (n), Leak (Omy), and non-mitochondrial, did not change (Figure 4E). Altogether, these results suggest that the altered mitochondrial morphology is associated with low mitochondrial ATP production in *EMC1*-silenced flies, which in turn could contribute to the low muscle performance shown here for these flies.

## 4. Discussion

Using *Drosophila melanogaster* as a model organism, we have determined that EMC1 function is essential for the striated muscle physiology, being required for SR and mitochondrial integrity. The data show the usefulness of *Drosophila* model to enlighten previously reported human neuromuscular diseases [1]. We show that EMC1 protein localizes to the SR and its depletion leads to a significant decrease in the density of the SR network and possibly an uneven accumulation of cisternae. Interestingly, a similar ER disorganization has been previously reported for *Drp1* [36] and *Mfn2* loss of function [37], two genes whose transcripts were significantly altered upon muscular *EMC1* silencing.

What is the underlying mechanism for the SR reduction/misdistribution associated to EMC1 depletion? It is possible that the EMC1 requirement for the maintenance of the SR network merely reflects its function as mediator of lipid exchange between the SR and mitochondria. It has been widely shown that lipid biosynthesis that occurs in the ER/SR–mitochondria interface requires exchange of lipids between these organelles in a mechanism dependent on close contact and tethering of their membranes [38,39,40,41]. This biosynthetic pathway is required for membrane expansion of the ER/SR and for whole cell growth [42]. Evidence for the requirement of the EMC complex as an additional tether of the ER to mitochondria, needed to mediate lipid transfer between these organelles, has been shown in yeast [18,19]. The data presented here are the first evidence for the requirement of EMC1 in the maintenance of SR morphology and mitochondrial function. The morphological alterations resulting from EMC1 depletion, not surprisingly, caused a major disturbance in the modulation of intracellular Ca^2+^ levels and could explain the phenotype of muscle failure. However, further studies are required to determine the primary defect, such as a physical loss of tethers, deficiency in lipid biosynthesis, and the origin of the defect in Ca^2+^ homeostasis.

Could reduction/misdistribution of the SR alone explain the mitochondrial alterations and deficient OXPHOS triggered by EMC1 depletion? SR disorganization in *EMC1*-silenced muscle might itself be the basis for the loss of mitochondrial integrity since phospholipid supply from the ER/SR to mitochondria is known to be required for integrity of the respiratory chain, energy production, and even mtDNA stability [42,43,44]. However, disruption of SR–mitochondrial and SR–myofibrils interfaces would impact the fine-tuned regulation of intracellular Ca^2+^, the *sine qua non* condition of excitation-contraction coupling in striated muscle leading to muscle failure. The decreased mitochondrial OXPHOS (Figure 4E) underscores the critical requirement for EMC1 to cope with the high-energy demand of the musculature. It has been well-demonstrated that Ca^2+^ propagation from the SR to mitochondria triggers the activation of ATP production to cope with the energetic demand of muscle contraction [44]. Efficient uptake of Ca^2+^ by mitochondria has also been shown to be dependent on ER–mitochondrial contact, requiring Mfn2 in non-muscle cells [37]. An alternative hypothesis for the deficient OXPHOS shown here is that ER stress due to primary accumulation of unfolded protein in the SR or as a reflex of increased levels in reactive oxygen species could contribute to the disorders observed. Indeed, we show evidence of high levels of cytosolic Ca^2+^ in silenced muscles concomitant to the high expression of ER-stress sensor genes and *CaMKII*, known to be upregulated/activated upon oxidative stresses [45]. A cytosolic Ca^2+^ overload could explain mitochondrial alterations and much of the muscular deformations shown in embryos, larvae, and adults, as in *SERCA* mutants [46]. Also, oxidative stress perturbing ER–membrane calcium channels can lead to cytosolic and mitochondrial Ca^2+^ overload, with subsequent muscle failure [47]. 

Does EMC1 play a role in the regulation of mitochondrial biogenesis and dynamics? Mitochondria are highly dynamic organelles whose function relies on coordinated genetic programs that respond on demand to regulate the rates of mitochondrial biosynthesis, fusion, fission, their cytoskeleton-dependent transport, and degradation via autophagy [48,49,50]. The results shown here suggest that the mechanisms of mitochondria quality control and/or biogenesis are disturbed in EMC1-depleted muscles, with implications in cellular energy supply. This may be part of a response to the accumulation of damaged mitochondria. It has been shown that mitochondria with reduced membrane potential, as is the case in EMC1-depleted flies, are prevented from fusing to the mitochondrial network by different mechanisms [50]. These isolated mitochondria are prone to mitophagy, an essential process for elimination of dysfunctional mitochondria. However, the decreased extension of the SR network shown for EMC1-depleted animals could limit the formation of autophagosomes, thereby also limiting mitophagy. It has been shown that EMC components are required for autophagy [51]. Although some morphological features of mitochondria in EMC1-depleted muscles resemble those of *parkin* null mutants [52], EMC1-depleted flies show more severe motor deficiencies with a much earlier onset, at the larval stage. Also, life span is more severely shortened in *EMC1*-silenced flies than in *parkin* null mutants [53]. When compared to null mutants of *porin*, which encodes the major mitochondrial outer membrane protein—shown to interact with the EMC complex in yeast and mammals [4,54]—*EMC1* silencing in flies leads to similar morphological alterations in mitochondria and motor disabilities, though substantially more pronounced. Thus, future efforts to clarify the roles of EMC1–porin interaction and other interorganellar protein–protein interactions involving EMC in the fly musculature, should be worthwhile. In fact, many structural interorganellar links remain to be properly characterized [37,43]. 

Is the muscle global phenotype due to EMC1 depletion entirely explainable by loss of functions in the level of the SR–mitochondrial interface or are important additional roles involved? The EMC complex was first identified in a global functional screening in *S. cerevisiae*, as a protein complex of the ER membrane involved in the unfolded protein response, particularly required for the folding of transmembrane protein [4]. Subsequently, mammalian EMC was identified as a complex of ten components, connected to the ER-associated degradation (ERAD) network through interaction with UBAC2 and Derlin-2 [54]. Direct evidence for a role of EMC in muscle comes from studies in *C. elegans* demonstrating that EMC components are required for the proper delivery of ionotropic receptors to the membrane [12]. In addition to abnormal formation of ER, misfolding of rhodopsin and other multi-pass membrane proteins were observed in the photoreceptors of *Drosophila EMC3* mutants [42,43,44]. Interestingly, EMC members were required to enhance biogenesis of a mutant CFTR channel in yeast, and could therefore modulate cystic fibrosis severity [55]. Although we did not observe defects in the myofibril sarcomere patterning, the altered shape of actin filaments in the embryo musculature, irregular distribution of nuclei, and deformed fibers have similarities to the dysfunction of *l(2)efl*, the fly homolog of *CRYAB*, whose product was postulated to function as a chaperone for the intermediate filament proteins [56].

In conclusion, the results presented here underscore the requirement of *EMC1* to mitochondrial and ER/SR integrity, essential to muscle physiology.

## Figures and Tables

**Table 1:** Sequences of the forward (F) and reverse (R) primers used for RT-qPCR analysis.

Primer Name	Primer Sequence (5′ 3′)	Reference
mtDNA	F-CAACCATTCATTCCAGCCTT R-GAAAATTTTAAATGGCCGCA	[25]
Dm_ACT79B	F-CCACGCCATCCTTCGTCTA R-GCACAGCTTCTCCTTGATGTC	[25]
EMC1	F-GCAAGGCCGGGGATTTCACA R-ATGTTATCGGACTGGGTCCT	-
EMC3	F-CAGGACGGCCAGGCAATGAT R-CGCCCGTTTCTGAGTCTTGAA	-
EMC5	F-ACAAACTCTTGCTAATTGCCGG R-TCTGCAGGATAATGTCCAATGG	-
EMC6	F-CTGCGCTGCGGGTATTTTGG R-CACTGGGTGCCCGATTTCAC	-
EMC8-9	F-TTTCCACCAGTGCCTGTATG R-TGCGTAGTAGCCTGCGATTA	-
IRE1	F-TGATGGTGTTCTCCACACTG R-TAAATGCTGCCATCTCGAGG	-
PERK	F-ATACTTCCATTCCTGGACCG R-AAGAGCTGCTGTTGGCGTTT	-
ATF6	F-AGAGTCTGCTTCCTTATCAC R-TTGCATTTCCAGATTCACGC	-
SPARGEL	F-GGATTCACGAATGCTAAATGTGTTCC R-GATGGGTAGGATGCCGCTCAG	[26]
MARF	F-GGCGAGGCGTATCTTATGAC R-AGCTTCTCCTGGCACAA	[27]
OPA1	F-CTCTGAGCACCAAGCTAT R-GGCGCAACTTGATGTCTA	[27]
DRP1	F-TCCATCCAATTGCCCCAAAT R-GACGGGTCACAATACCAGTT	-
PINK	F-GCTTTCCCCTACCCTCCAC R-GCACTACATTGACCACCGATT	[28]
PARKIN	F-AGCCTCCAAGCCTCTAAATG R-CACGGACTCTTTCTTCATCG	[29]
CAMKII	F-CCTGTACGCGTTTTTCGGAC; R-CCTCCTGTATACTGTCATGT	-
RPL32A	F-ATGCTAAGCTGTCGCACAAATG R-GTTCGATCCGTAACCGATGT	[30]
EF1	F-GCGTGGGTTTGTGATCAGTT R-GATCTTCTCCTTGCCCATCC	[30]

## Data Availability

Data are contained within the article.

## Supplementary Materials

The following supporting information can be downloaded at: https://www.mdpi.com/article/10.3390/biom14101258/s1, Figure S1: Detection of the EMC1 polypeptide and transcripts; Figure S2: Overexpression of *EMC1* in the *Drosophila* musculature; Figure S3: Altered sarcoplasmic reticulum and mitochondrial morphology upon changes in *EMC1* transcripts levels; Video S1: *Drosophila* eclosion; Video S2: EMC1-silenced flies have severe difficulty in climbing the walls of their vials; Video S3: Overexpression and rescue in the *Drosophila* eclosion; Video S4: EMC1-rescue flies climbing the walls of their vials.
