# Supplementary material for: EMC1 Is Required for the Sarcoplasmic Reticulum and Mitochondrial Functions in the Drosophila Muscle
Source: Biomolecules. 2024 Oct 5;14(10):1258. doi: 10.3390/biom14101258 (PMC11506464; doi:10.3390/biom14101258)
Supplement: Supplementary file 1 [file biomolecules-14-01258-s001.zip › Supplementary figure and movie legends.pdf]

SUPPLEMENTAL FIGURE LEGENDS

Supplemental Figure S1. Detection of the EMC1 polypeptide and transcripts. (A) Schematic representation of the EMC1 polypeptide in humans and *Drosophila*, showing the predicted domains (SP, ER-targeting signal peptide; PQQ, pyroloquinolinequinone-binding domains; ZL, leucine-zipper domain; DUF1620, unknown function) and the region (460 to 915 aa) of the fly protein that was fused to a 6xHis-tag for antibody development (see main text for details). **(B)** Immunoblots against the recombinant fused protein expressed in bacterial cells using a monoclonal anti-His antibody (left panel), and against the full-length native EMC1 protein in extracts of w^{1118} embryos, L3 and adult thoraces using the polyclonal anti-EMC1 antibody developed here (right panel). **(C)** *EMC1* transcript levels were calculated as described in the Material and Methods for w^{1118} individuals of the indicated developmental stage. Transcripts were normalized by the levels of *EMC1* in third-instar larvae (L3). **(D)** Representative images of 2-day-old female adults of the *Mef2>+* (control) and *Mef2>EMC1 RNAi* V8477 (*EMC1*-silenced) genotypes that were fed food supplemented with the methylene blue dye for 24 hours prior to the analysis. **(E)** Dissected intestines of the same animals, indicating no food consumption for *EMC1*-silenced adults. **(F)** Representative wings of 2-day-old flies, showing malformations commonly found in *EMC1*-silenced animals. Some of these animals completely failed to expand their wings, others showed curled wings and yet others showed wings of shrunken appearance (data not shown).

Supplemental Figure S2. Overexpression of *EMC1* in the *Drosophila* musculature. Relative *EMC1* transcript levels in the newly generated *UAS-EMC1-OE* lines **(A)** and in combination with the *UAS-EMC1-RNAi* construct **(B)**. Note in **A** that induction of *EMC1* overexpression was inefficient for the new transgenic line 22.2. **(C)** Immunoblot (upper panel) and band densitometry

(lower panel) showing EMC1 polypeptide levels in the indicated *EMC1* lines. **(D)** The adult eclosion process is represented from frames of movies recorded for *Mef2*>+ control, *Mef2>EMC1-RNAi*, *Mef2>EMC1-OE* and *Mef2>EMC1-OE;EMC1-RNAi* individuals. The initial time represents the first adult movements still inside the puparium, and the final times are when the animals leave the pupal case or notably fail to do so (see details in Movies S1 and S3). **(E)** Adult life span analyses were performed as described in the Materials and Methods, using individuals of the indicated genotype. **(F)** Representative images of adult flies of the indicated genotype. **(G)** Quantitation (average +/-S.D.) of the indicated physical feature of the flies shown in **F**. **, *** and **** indicate respectively $p<0.01$, $p<0.001$ and $p<0.0001$, according to Student´s t-tests applied between the *Mef2*>+ controls and *Mef2>EMC1-RNAi*, *Mef2>EMC1-OE* or *Mef2>EMC1-OE;EMC1-RNAi* animals.

Supplemental Figure S3. Altered sarcoplasmic reticulum and mitochondrial morphology upon changes in *EMC1* transcripts levels. (A) Immunofluorescent confocal microscopy images of representative indirect flight muscles of *Mef2*>+ and *Mef2>EMC1-RNAi* eclosed adults and arrested pupae (1 day passed eclosion), stained with anti-Calreticulin and marked with mitoGFP. Levels of mitochondrial DNA **(B)** and transcripts of the indicated genes **(C-E)** were calculated as described in the Material and Methods for individuals of the indicated genotypes and age, normalized by the levels in the *Mef2*>+ control (dashed red lines). **(F)** mitoGFP signal of adult musculature of the indicated genotype. * and ** indicate respectively $p<0.05$ and $p<0.01$, according to Student´s t-tests applied between *Mef2*>+ controls and *Mef2>EMC1-RNAi* or *Mef2>EMC1-OE;EMC-RNAi* animals.

SUPPLEMENTAL MOVIE LEGENDS

Movie S1: Drosophila Eclosion. (A) Control fly. Continuous Movie, recorded from the initial pre-eclosion movements (00:00:00 - 00:01:03) to the actual eclosion (00:01:03 - 00:01:31) from the puparium, which took only about 30 sec. **(B-D)** EMC1-silenced flies exhibiting phenotypes with various degrees of severity. Flashes of the eclosion events subdivided in three periods: (B) Initial movements pre-eclosion (00:00:00 to 00:01:00), rupture of the pupal case (00:35:00 to 00:35:30 + 02:10:30 to 02:11:00), and actual eclosion (02:58:05 - 02:59:05); (C) Initial movements pre-eclosion (00:00:00 - 00:01:00), rupture of pupal case (00:42:51 to 00:43:51), and the end of the Movie (5:58:03 - 06:00:07), showing failure of eclosion; (D) initial movements pre-eclosion (00:00:00 - 00:01:00 interval), intermediate time interval (07:00:00 to 07:01:00) and the end of the Movie (13:58:00 to 14:00:00). The latter shows a more severe phenotype with failure even to rupture the pupal case.

Movie S2: EMC1-silenced flies have severe difficulty in climbing the walls of their vials.
(A) Control (left) and EMC1-silenced (right) flies. **(B)** A closer view of the movie.

Movie S3: Overexpression and rescue in the Drosophila eclosion.
(A) Control fly. Continuous Movie, recorded from the initial pre-eclosion movements (00:00:00 - 00:01:03) to the actual eclosion (00:01:03 - 00:01:31) from the puparium, which took only about 30 sec. **(B)** EMC1-OE flies, initial movements pre-eclosion (00:00:00 to 00:00:30), rupture of the pupal case (00:00:35 to 00:02:00), and actual eclosion (00:02:00 - 00:03:10); **(C)** EMC1-rescue

flies, initial movements pre-eclosion (00:00:00 - 00:00:30), rupture of pupal case (00:03:28 to 00:04:57), and actual eclosion (00:07:00 - 00:08:15).

Movie S4: EMC1-Rescue flies in climbing the walls of their vials. **(A)** Control flies **(B)** Overexpressed flies and **(C)** Rescue flies
