# Supplementary figures and images for: EMC1 Is Required for the Sarcoplasmic Reticulum and Mitochondrial Functions in the Drosophila Muscle
Source: Biomolecules. 2024 Oct 5;14(10):1258. doi: 10.3390/biom14101258 (PMC11506464; doi:10.3390/biom14101258)

**A*****Homo sapiens* EMC1**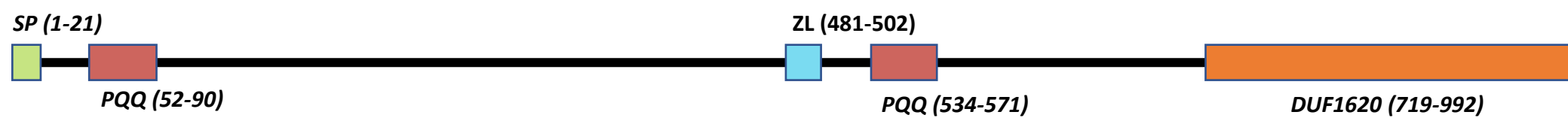***Drosophila melanogaster* EMC1**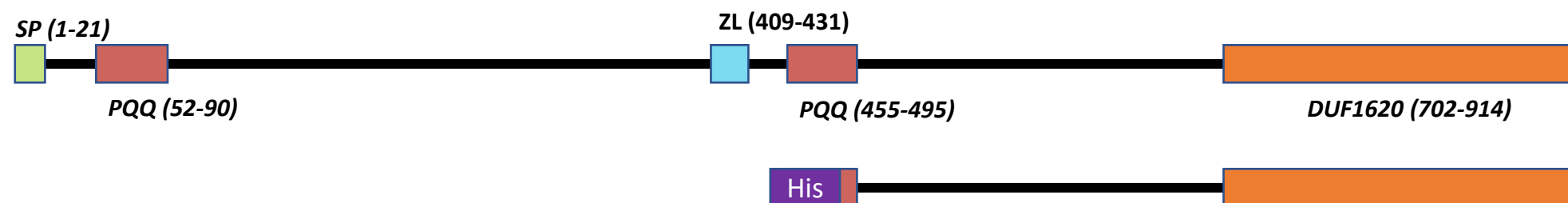**B**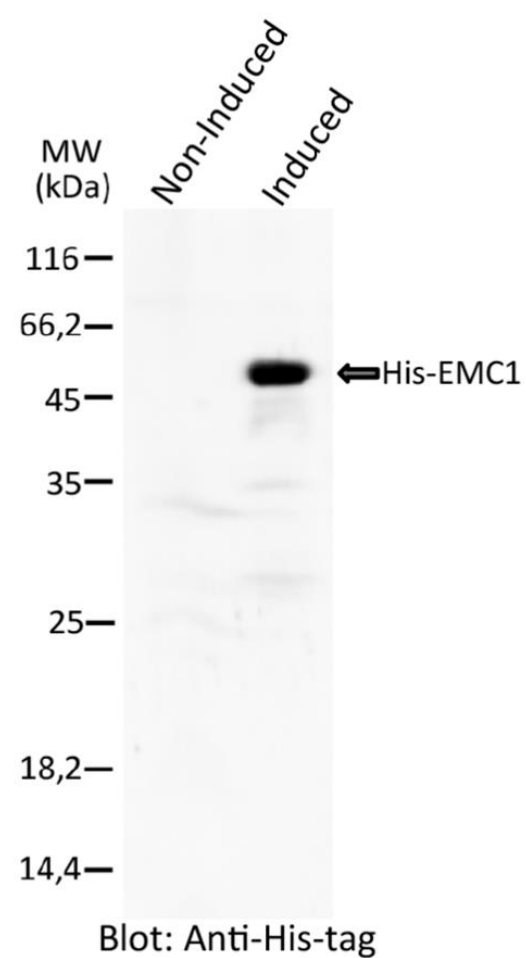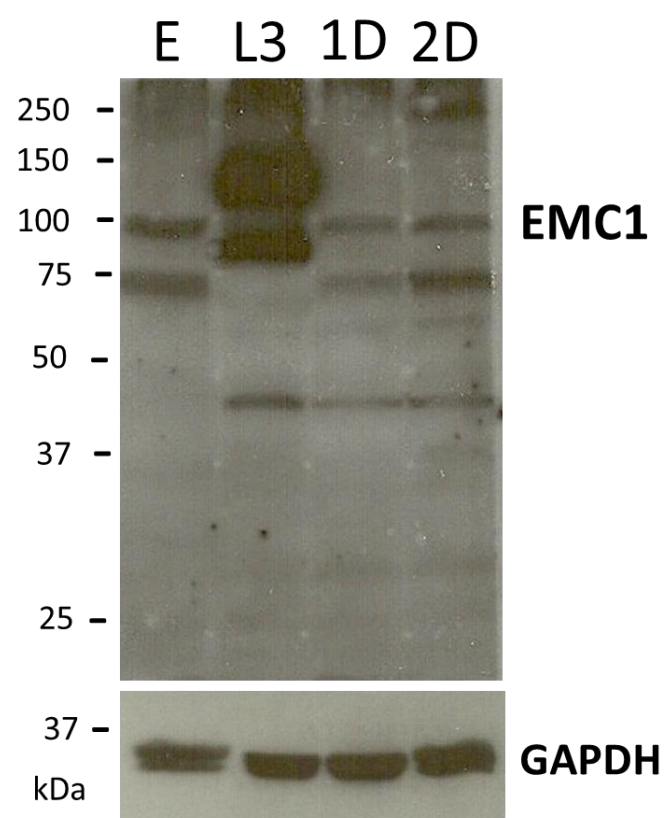**C**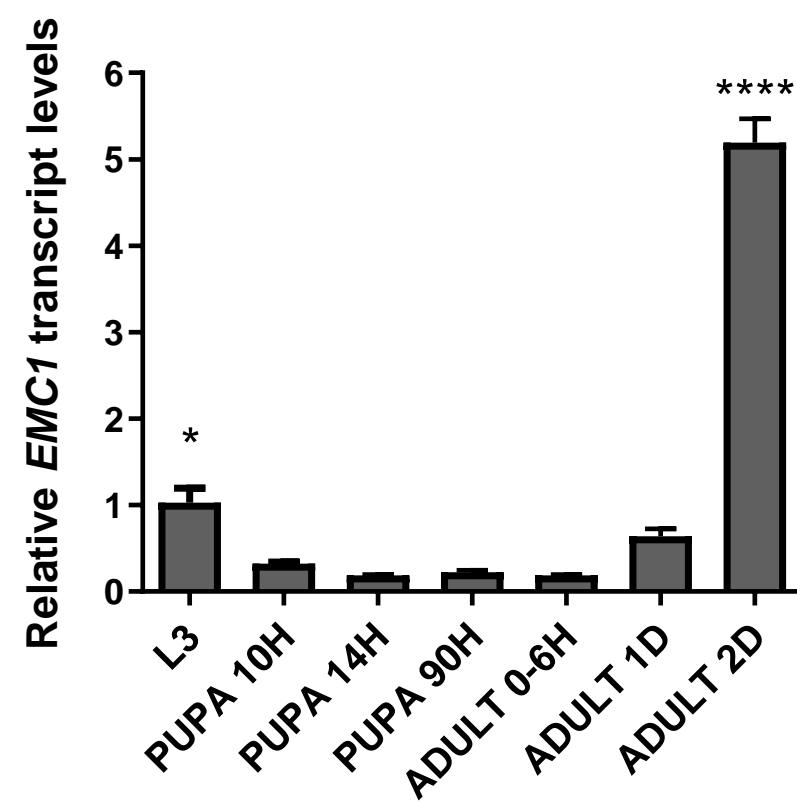**D**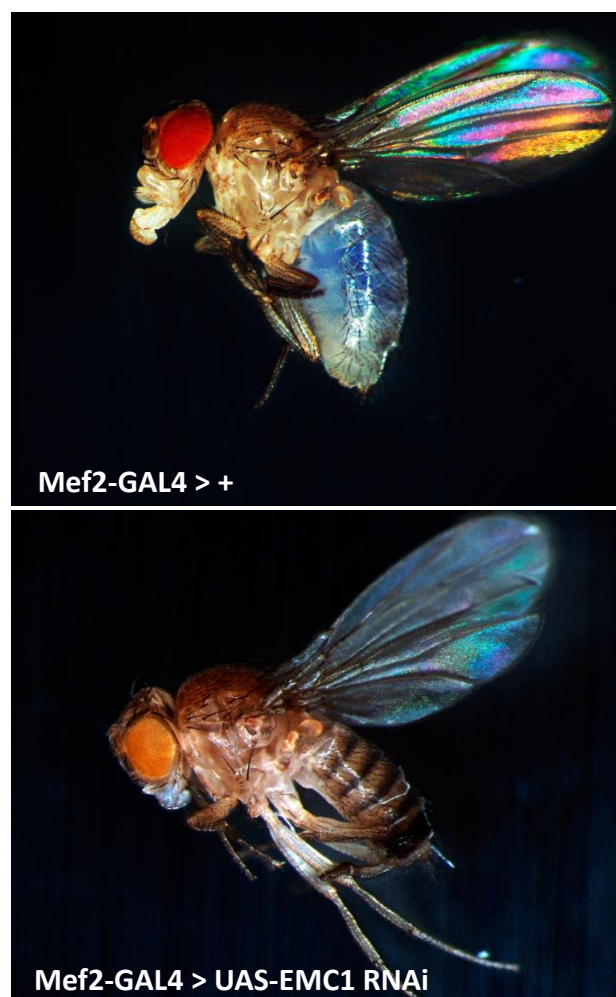**E**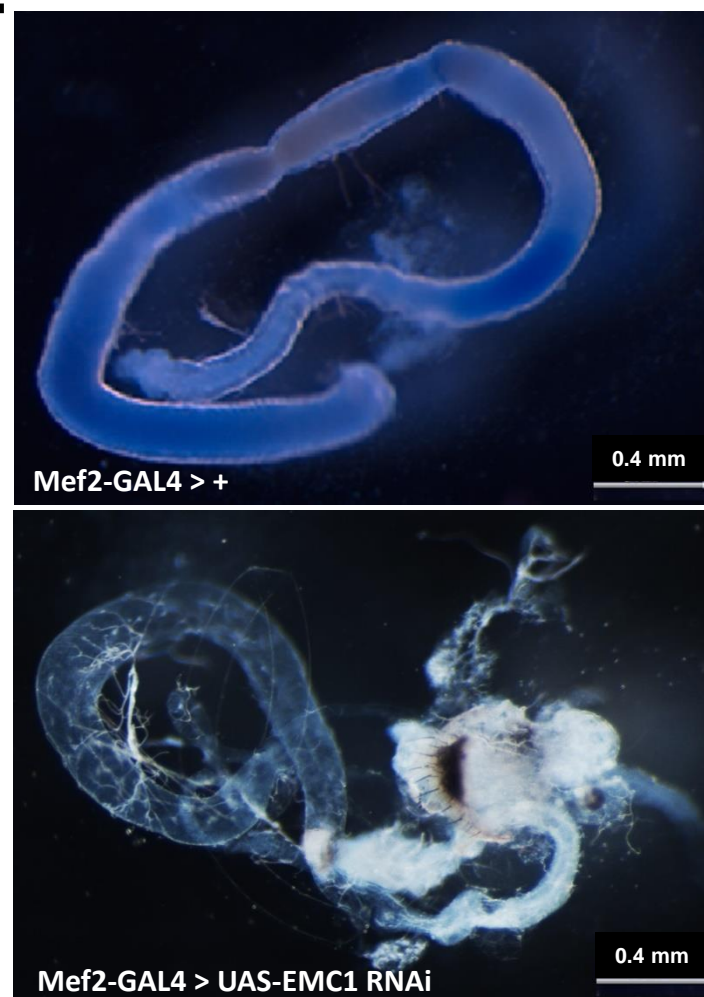**F**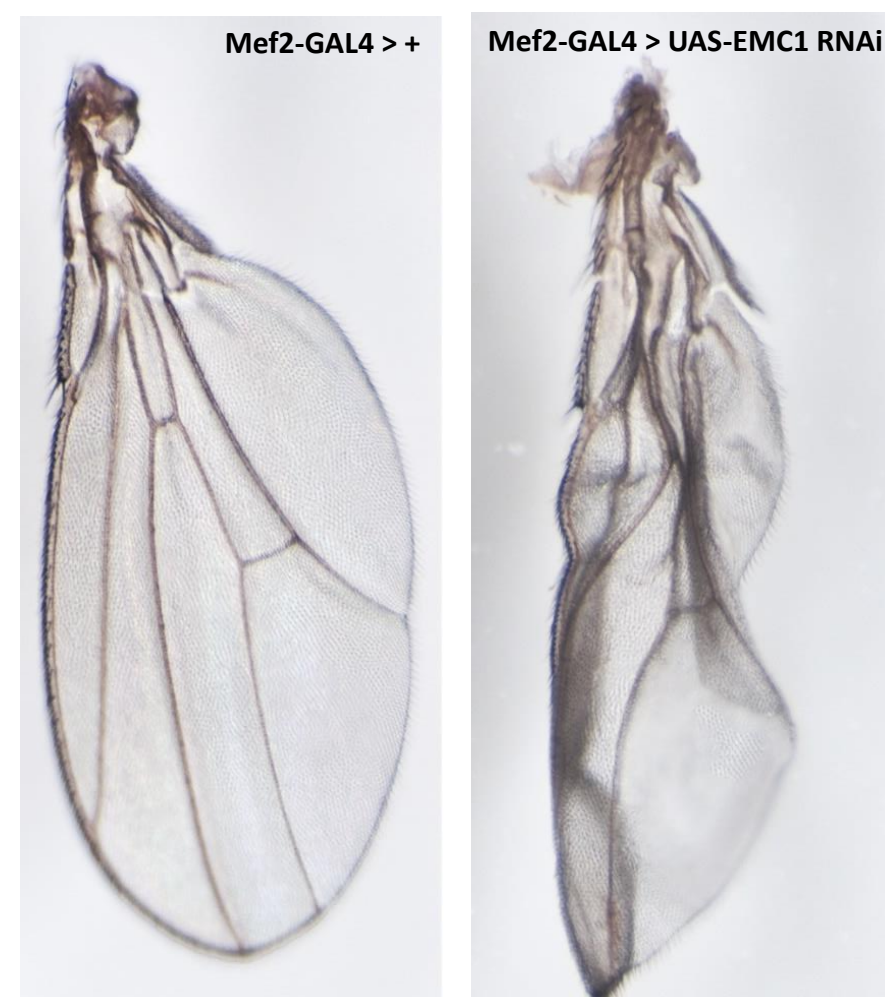

Supplement: Supplementary file 1 [file biomolecules-14-01258-s001.zip › Supp Fig S1.pdf]

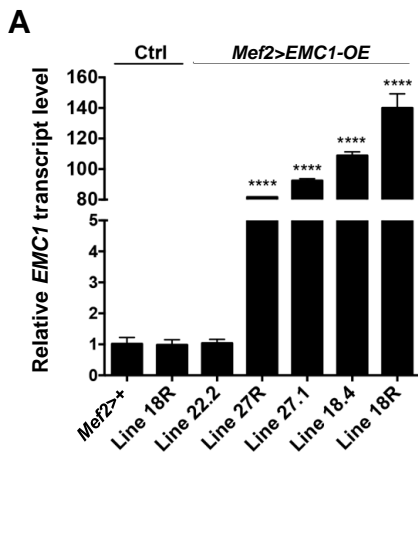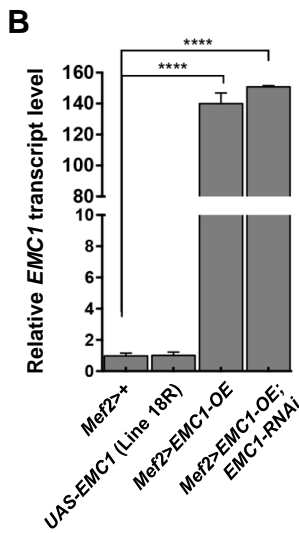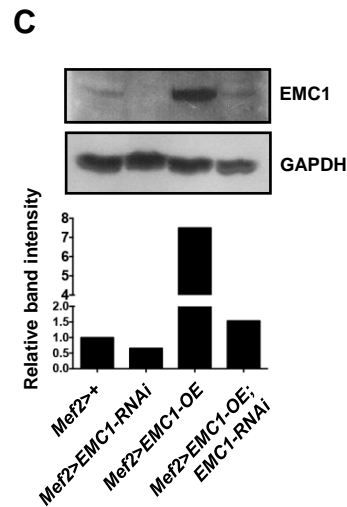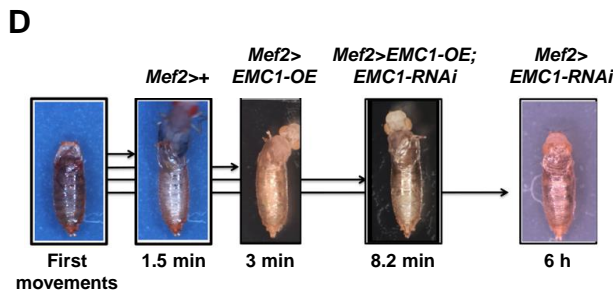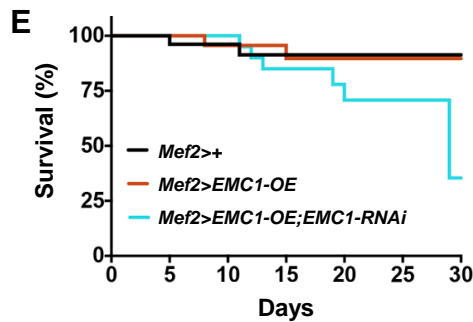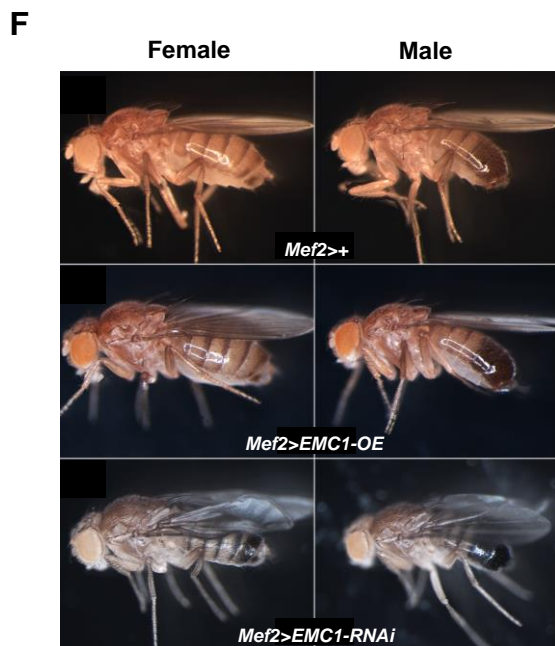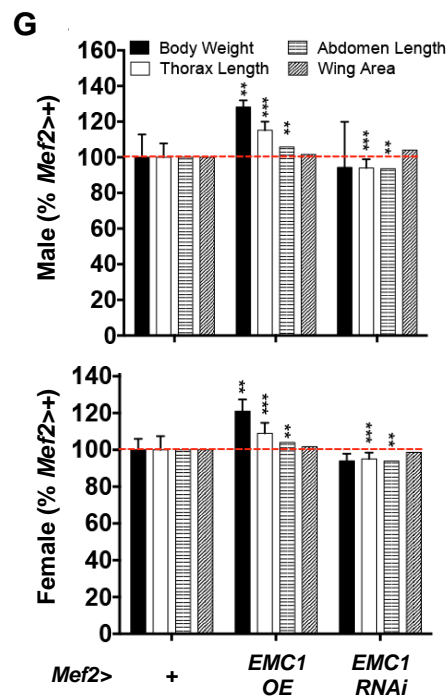

Supplement: Supplementary file 1 [file biomolecules-14-01258-s001.zip › Supp Fig S2.pdf]

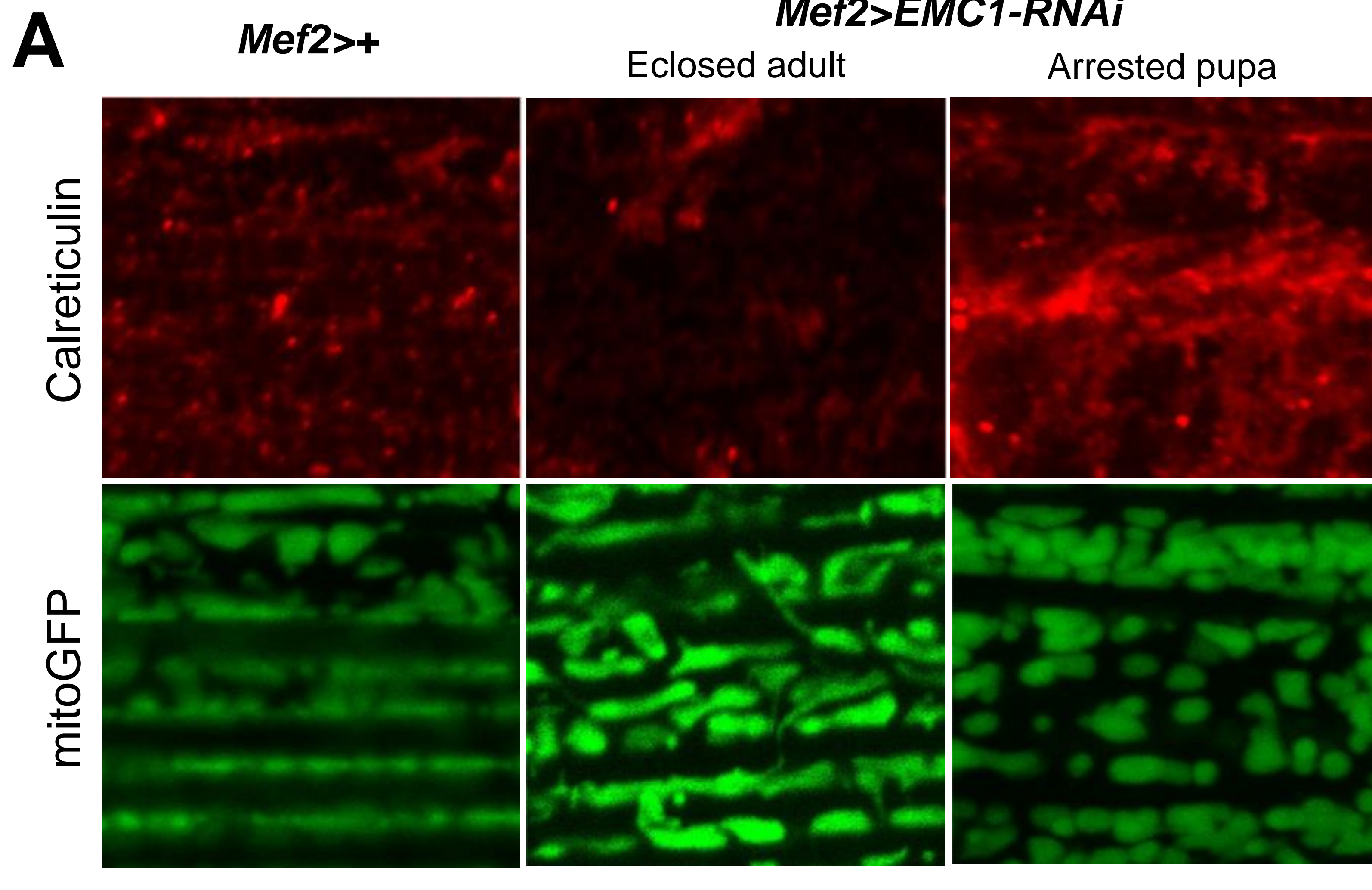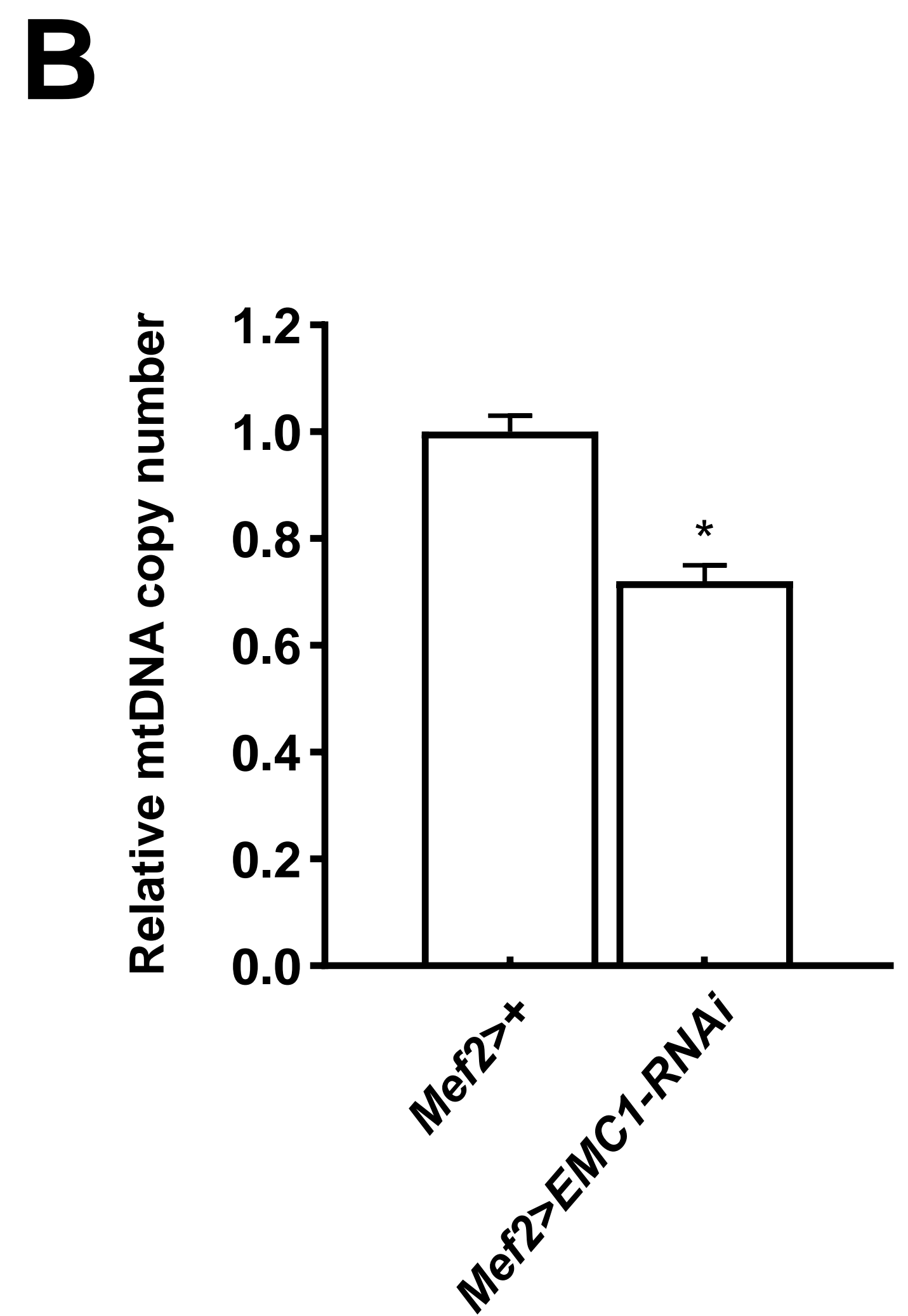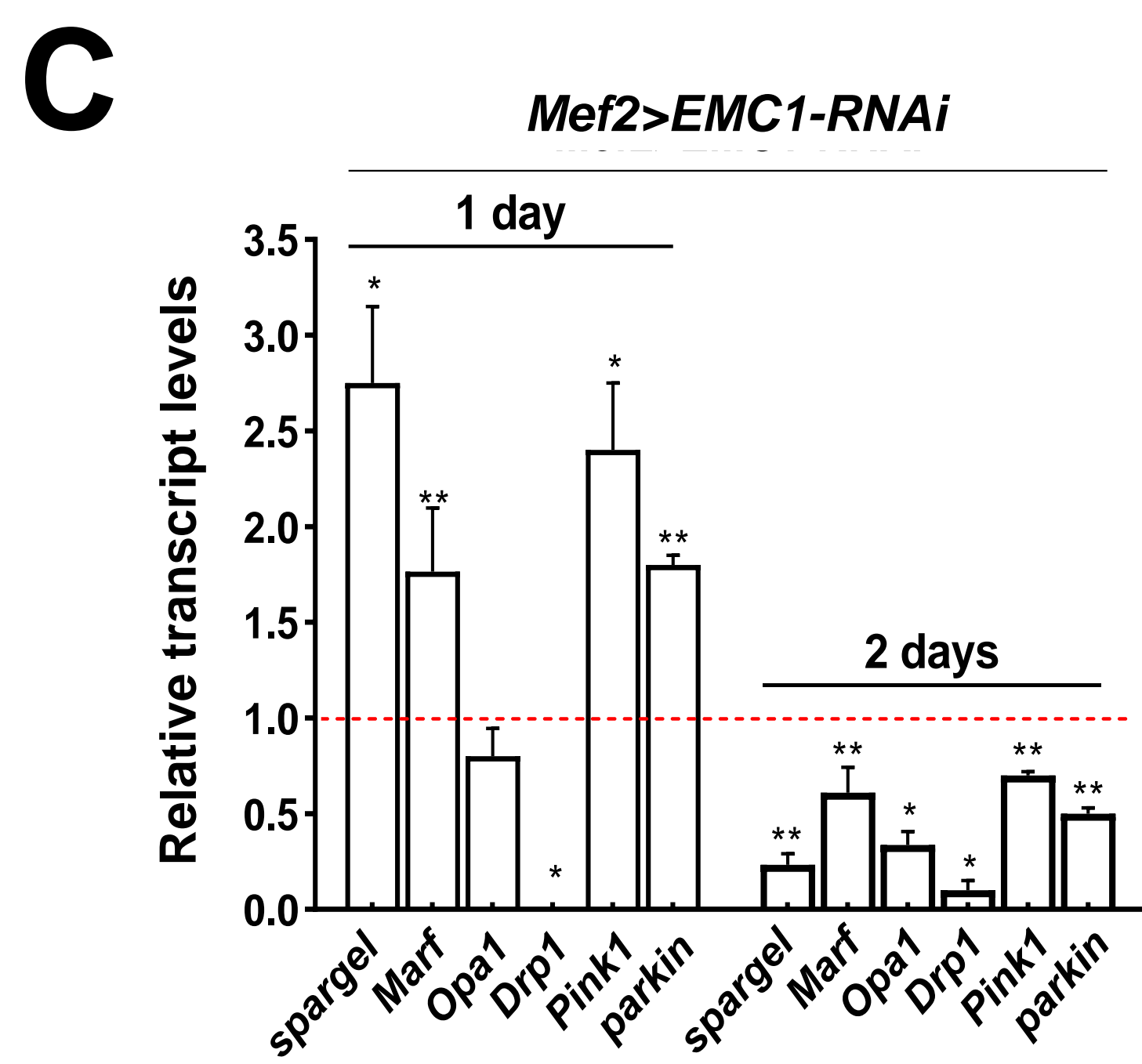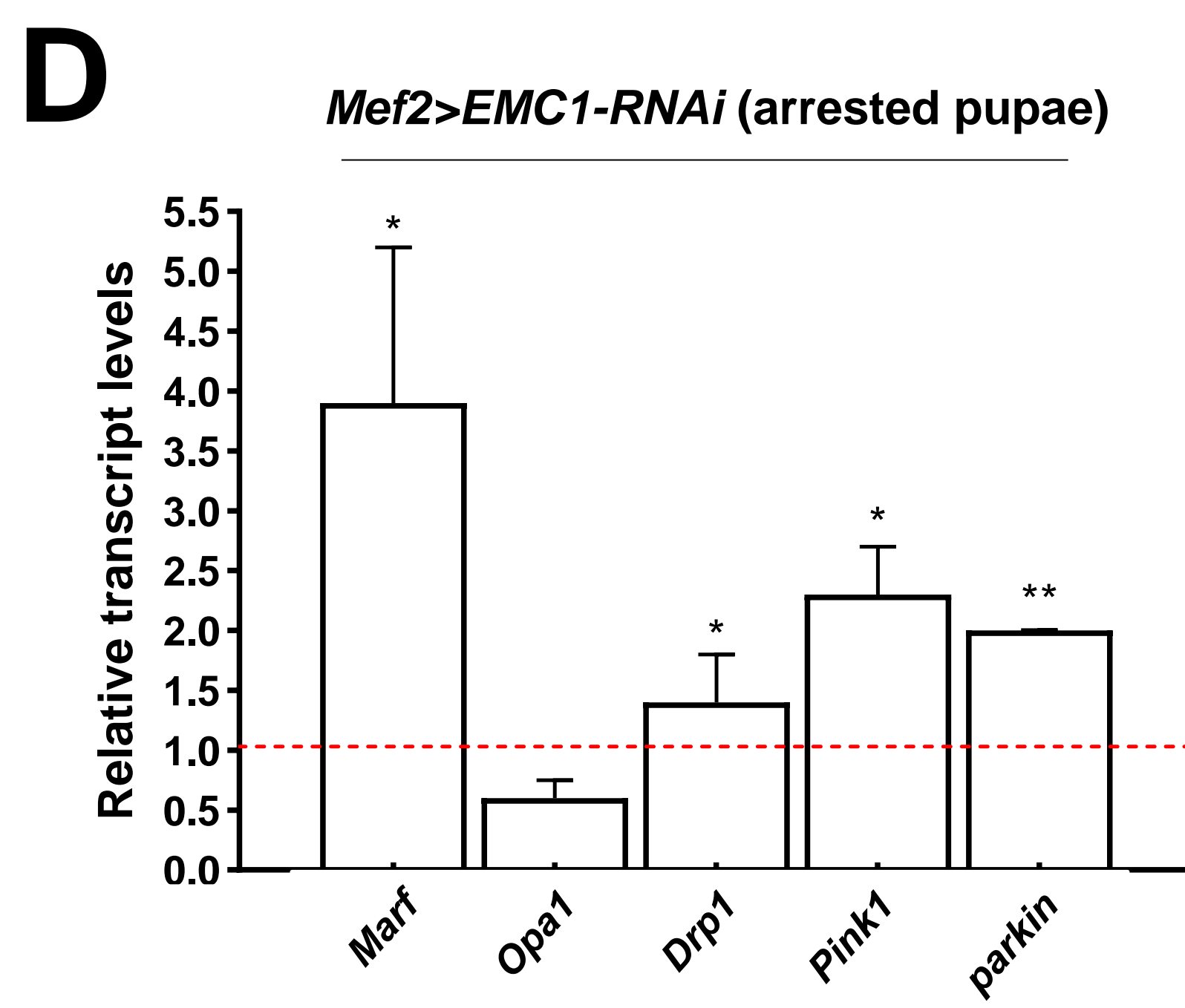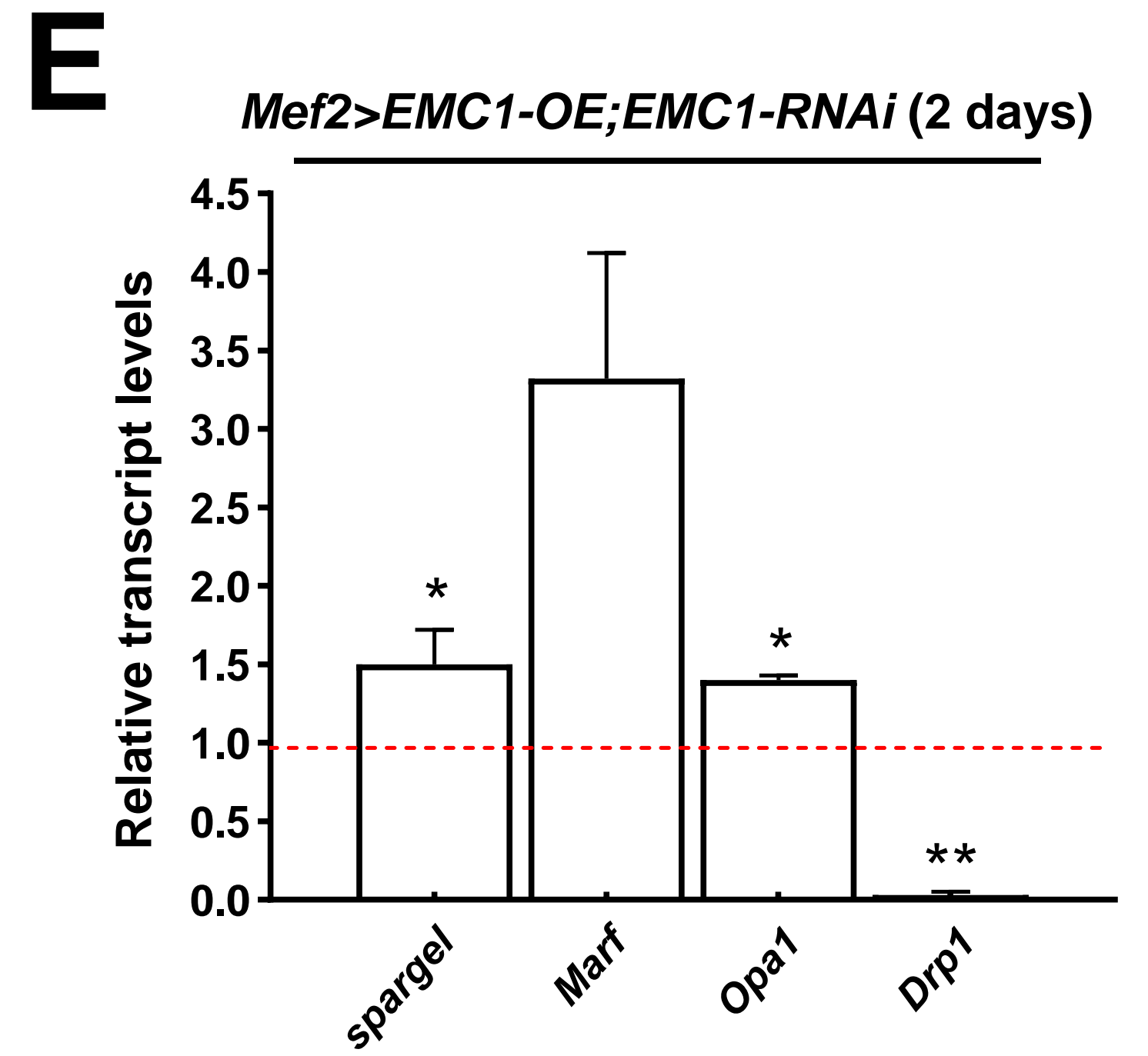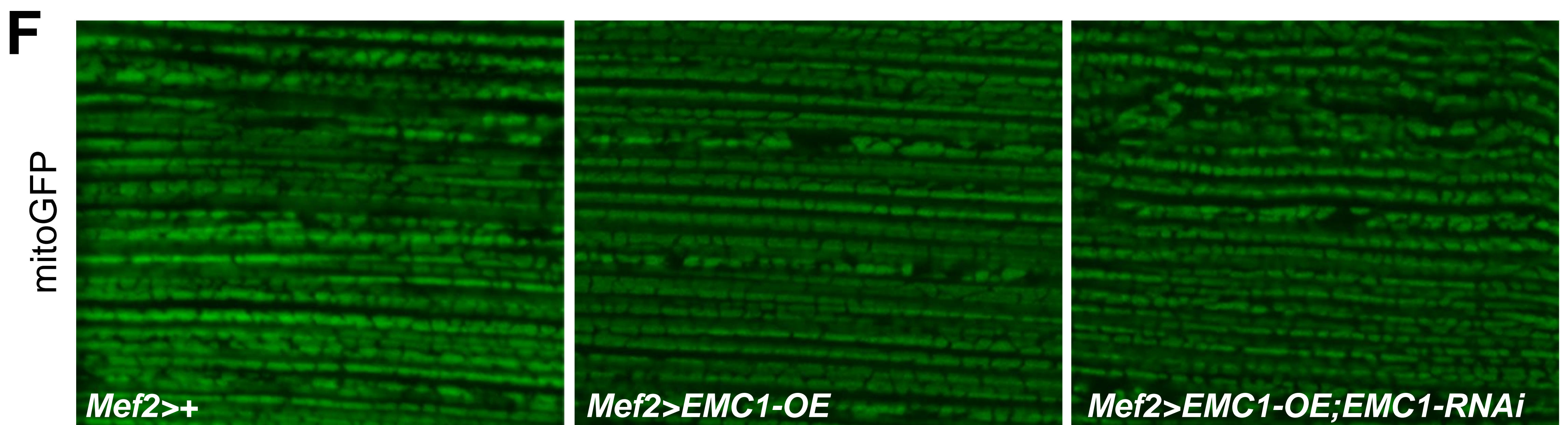

Supplement: Supplementary file 1 [file biomolecules-14-01258-s001.zip › Supp Fig S3.pdf]
